# Supplementary material for: Single-Trial Decoding of Scalp EEG under Natural Conditions
Source: Comput Intell Neurosci. 2019 Apr 17;2019:9210785. doi: 10.1155/2019/9210785 (PMC6501266; doi:10.1155/2019/9210785)
Supplement: Supplementary 2 — Figures S1–S10 contain supplementary material and are used for reference in the main manuscript. [file 9210785.f2.pdf]

Supplementary materials

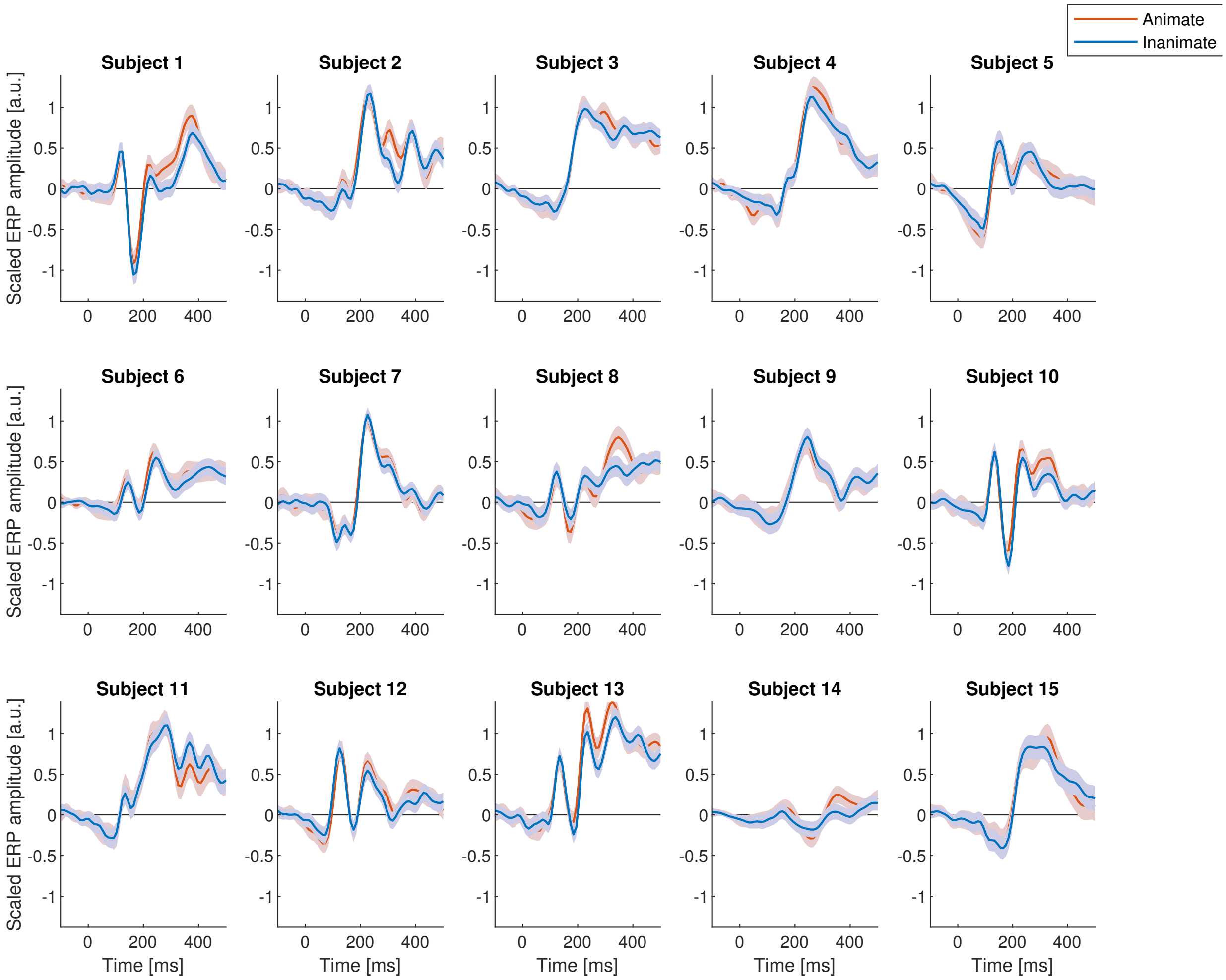

Fig. S1. Animate and inanimate ERPs for each subject separately with two standard errors around the mean.

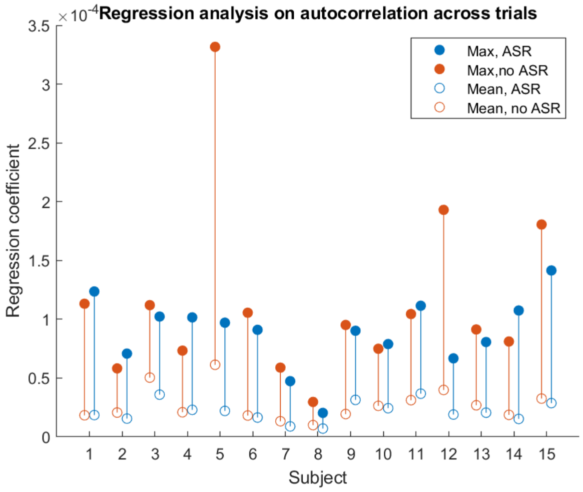

Fig. S2. Time dependency as quantified by autocorrelation. A high regression coefficient means that a channel (or an average of all channels) had an autocorrelation which increased or decreased linearly with time lags, and is thus indicative of high time dependency.

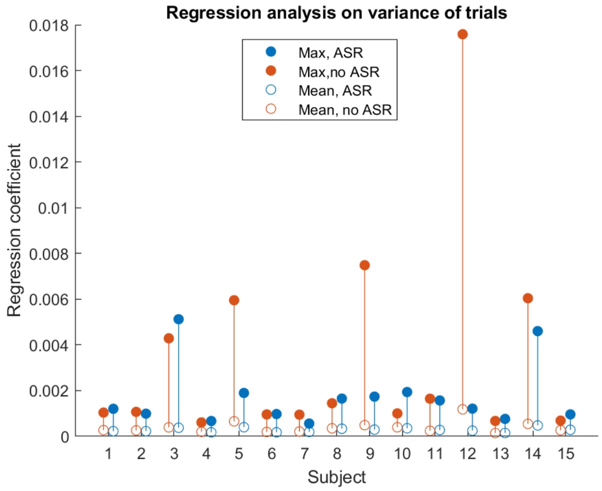

Fig. S3. Time dependency as quantified by variance of trials. A high regression coefficient means that a channel (or an average of all channels) had a trial variance which increased or decreased linearly with trial number, and is thus indicative of high time dependency.

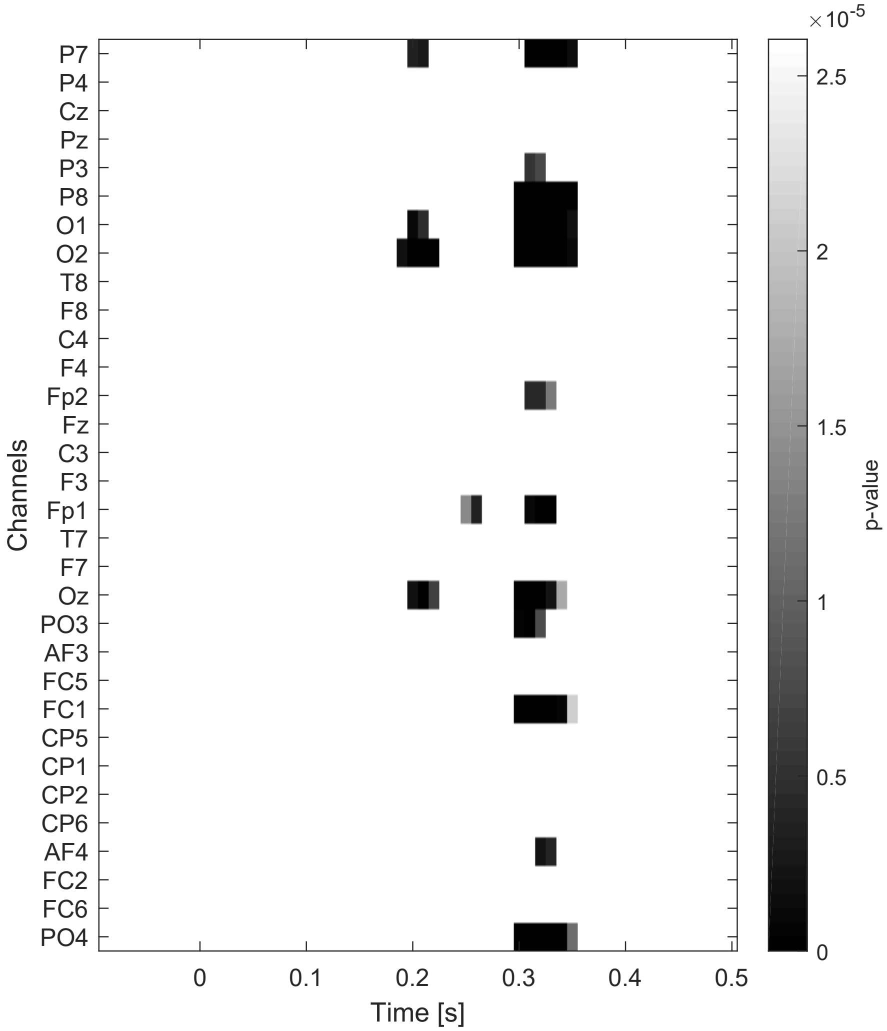

Fig. S4. Significant ERP differences between animate and inanimate trials (all subjects). Significance tested using a paired t-test. Figure thresholded at $\alpha = 0.05/(60 \cdot 32)$, i.e. Bonferroni corrected for multiple comparisons.

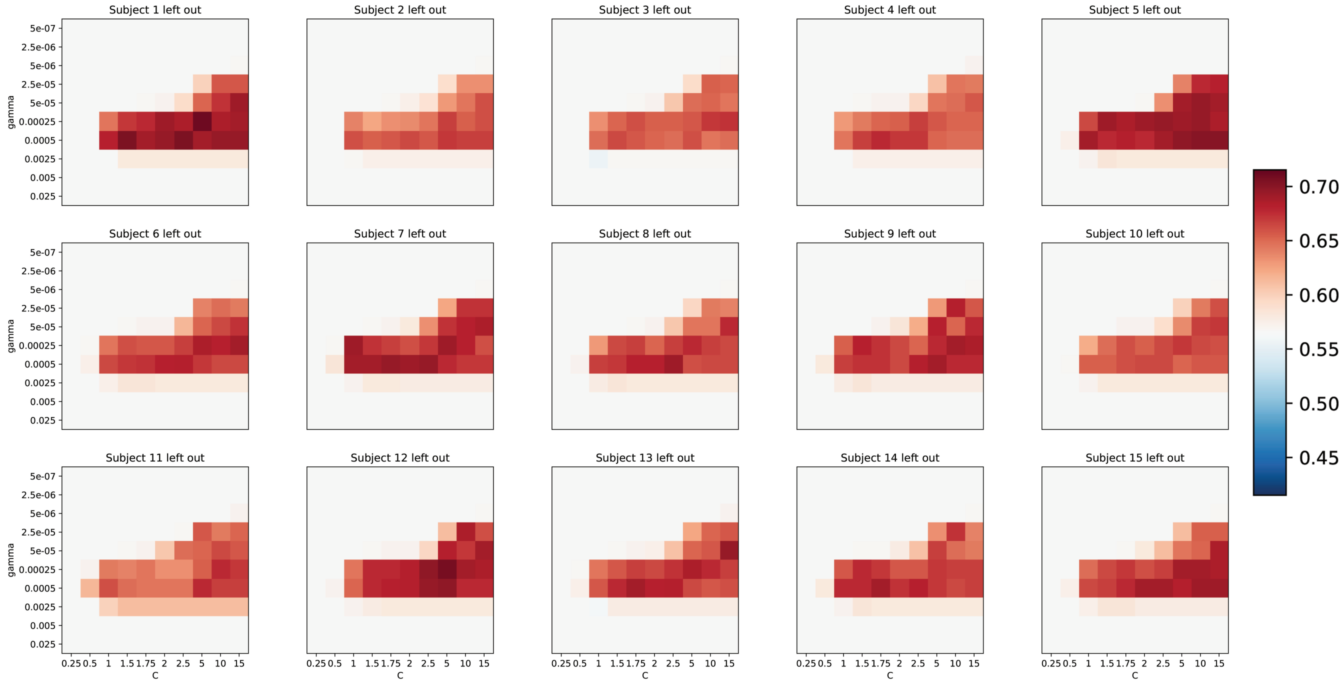

Fig. S5. Validation accuracies (mean over validation sets) for the pseudo-trial classifier. c values are displayed on the x-axis, and consisted of values: $[0.25, 0.5, 1, 1.5, 1.75, 2, 2.5, 5, 10, 15]$. γ values are displayed on the y-axis, and consisted of values: $[5 \times 10^{-7}, 2.5 \times 10^{-6}, 5 \times 10^{-6}, 2.5 \times 10^{-5}, 5 \times 10^{-5}, 2.5 \times 10^{-4}, 5 \times 10^{-4}, 2.5 \times 10^{-3}, 5 \times 10^{-3}, 2.5 \times 10^{-2}]$. Same scaling for all subjects.

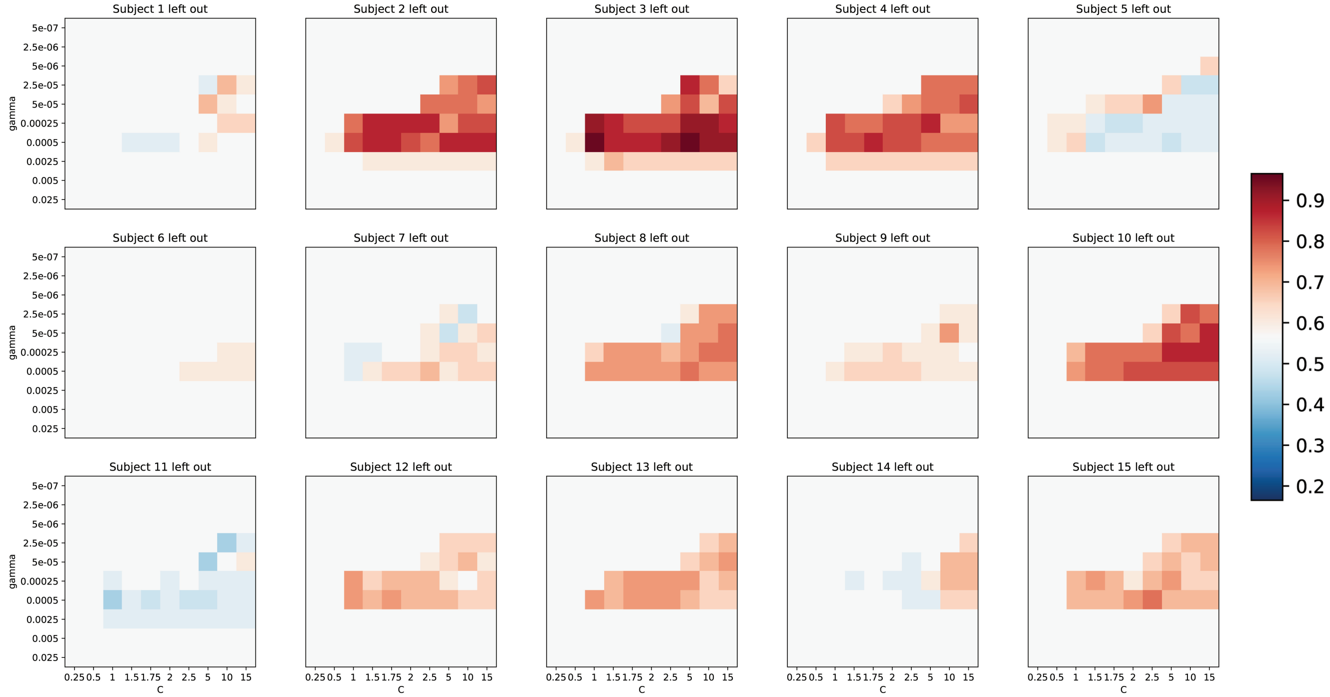

Fig. S6. Test accuracies for pseudo-trial classifier. Tested on the pseudo-trials (averaged categories) of the withheld subject. Same cross-validation parameter values as in Figure S5.

.

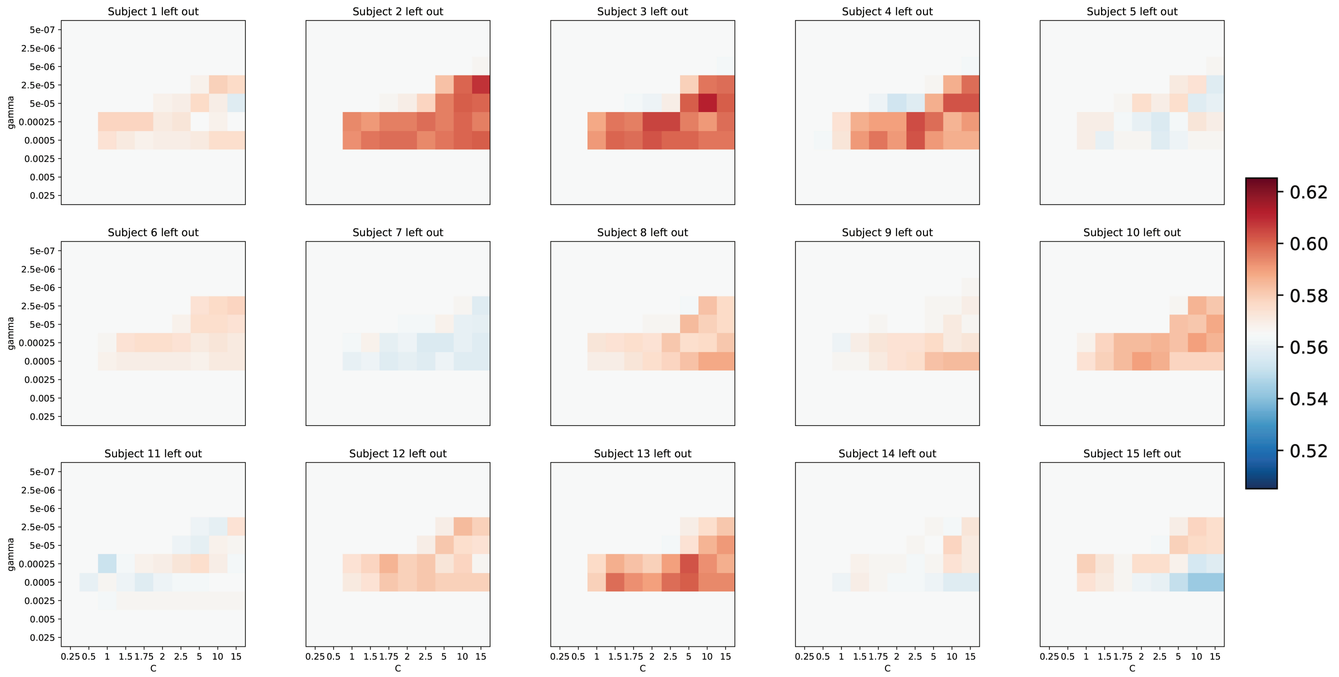

Fig. S7. Test accuracies for pseudo-trial classifier. Tested on the pseudo-trials (averaged categories) of the withheld subject. Same cross-validation parameter values as in Figure S5.

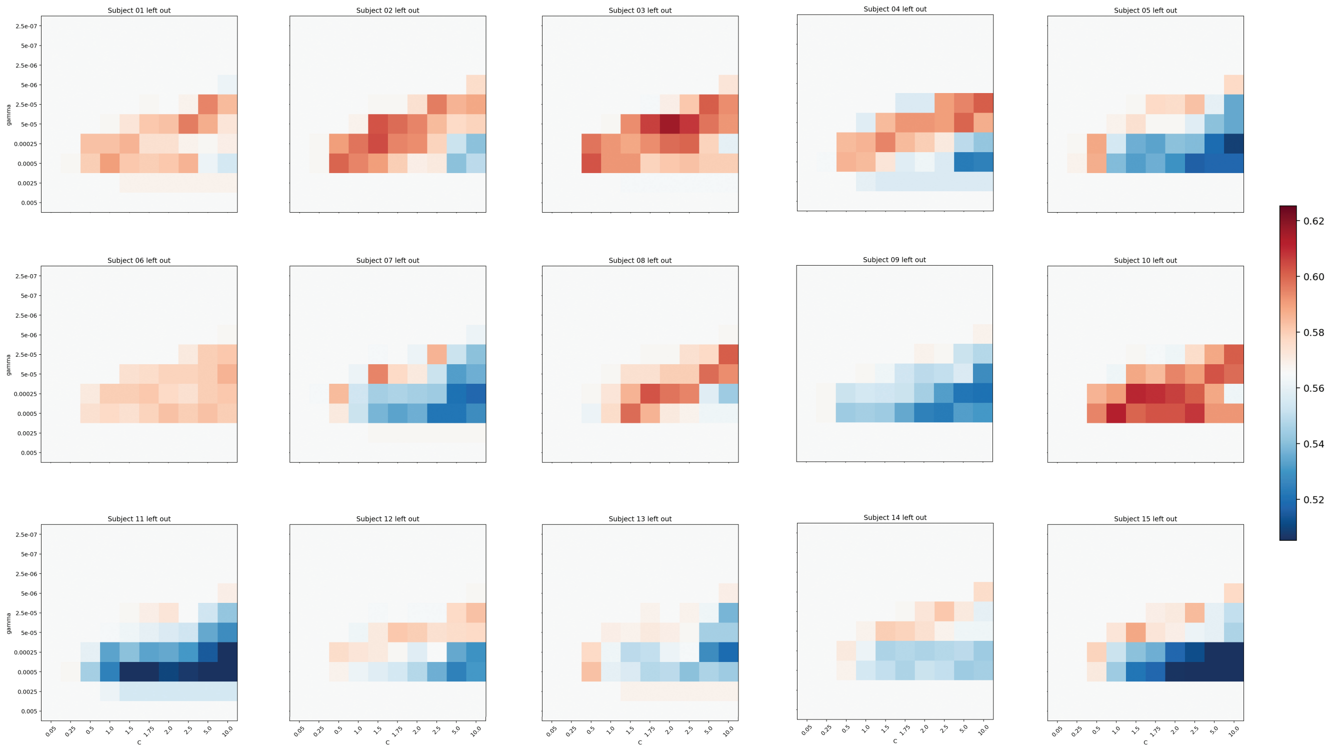

Fig. S8. Cross-validation with a single held out subject to estimate parameters for the upper level performance single-trial SVM classifier. c values are displayed on the x-axis, and consisted of values: $[0.05, 0.25, 0.5, 1, 1.5, 1.75, 2, 2.5, 5, 10]$. γ values are displayed on the y-axis, and consisted of values: $[2.5 \times 10^{-7}, 5 \times 10^{-7}, 2.5 \times 10^{-6}, 5 \times 10^{-6}, 2.5 \times 10^{-5}, 5 \times 10^{-5}, 2.5 \times 10^{-4}, 5 \times 10^{-4}, 2.5 \times 10^{-3}, 5 \times 10^{-3}]$.

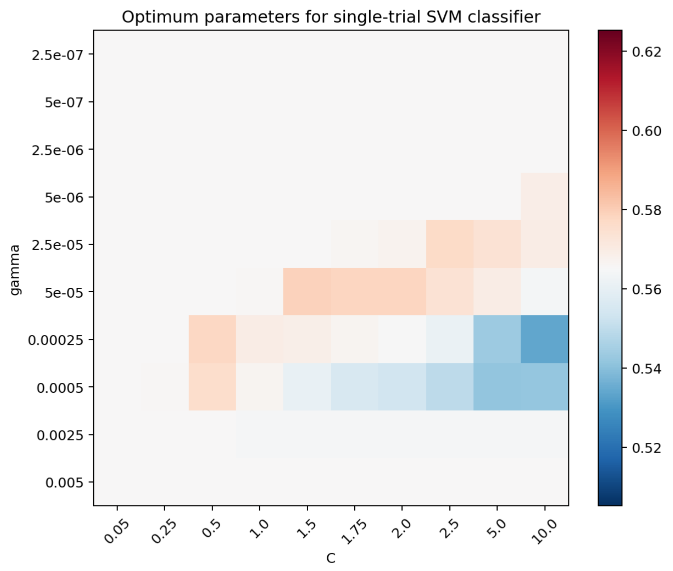

Fig. S9. Upper level performance parameters for the single-trial SVM classifier based on the mean parameters for held out subjects in Figure S8.

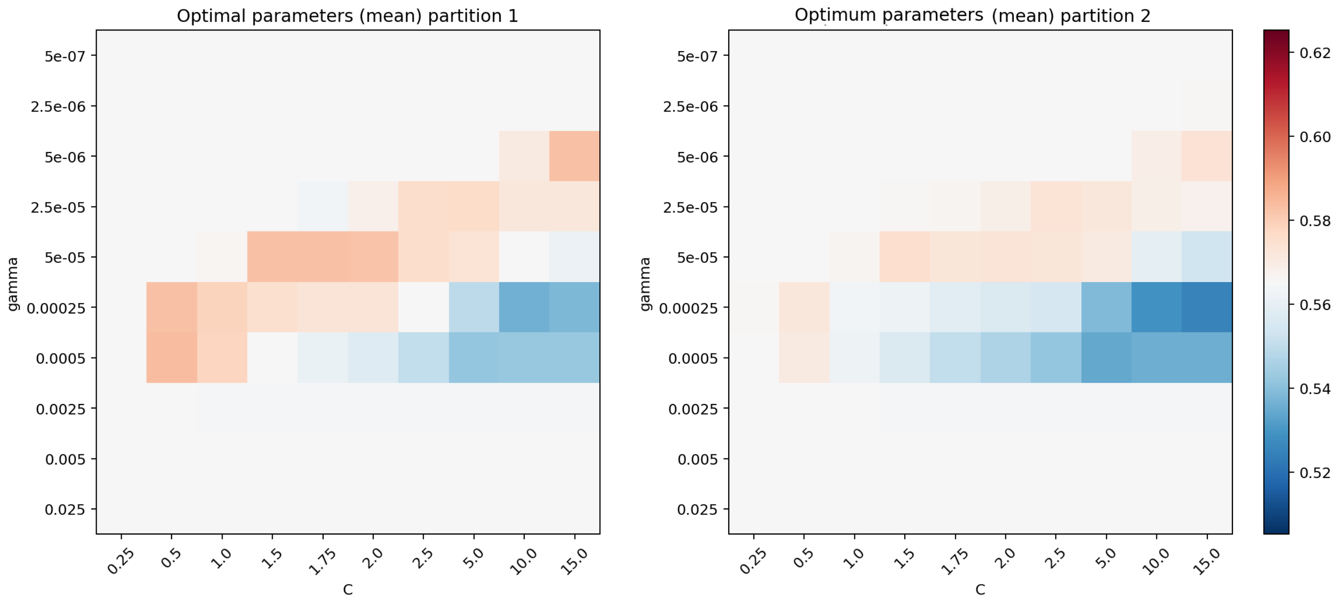

Fig. S10. Optimum parameters for the single-trial SVM classifier based on the mean parameters of validation partition 1 (subjects 1-7) and partition 2 (subjects 8-15). Same cross-validation parameter values as in Figure S5.
